# Slowly progressive autosomal dominant Alport Syndrome due to *COL4A3* splicing variant

**DOI:** 10.1038/s41431-024-01706-8

**Published:** 2024-10-19

**Authors:** Sergio Daga, Lorenzo Loberti, Giulia Rollo, Loredaria Adamo, Olga Lorenza Colavecchio, Giulia Brunelli, Kristina Zguro, Sergio Antonio Tripodi, Andrea Guarnieri, Guido Garosi, Romina D’Aurizio, Francesca Ariani, Rossella Tita, Alessandra Renieri, Anna Maria Pinto

**Affiliations:** 1 Medical Genetics, University of Siena, Siena, Italy; 2 Med Biotech Hub and Competence Center, Department of Medical Biotechnologies, University of Siena, Siena, Italy; 3 Genetica Medica, Azienda Ospedaliero-Universitaria Senese, Siena, Italy; 4 Institute of Informatics and Telematics, CNR, Pisa, Italy; 5 Department of Pathology, AOUS, Siena, Italy; 6 Department of Medical Sciences, Nephrology, Dialysis and Transplantation Unit, University Hospital of Siena, Siena, Italy

**Keywords:** Alport syndrome, Genetics research

## Abstract

Alport syndrome is a rare genetic kidney disease caused by variants in the *COL4A3/A4/A5* genes. It’s characterised by progressive kidney failure, though therapies targeting Renin-Angiotensin System can delay its progression. Additionally, extrarenal manifestations may sometimes coexist. Recent advances in genetic analysis and the necessity to better clarify genotype-phenotype correlations in affected patients raises the importance of detecting even cryptic splicing variants, lying in both canonical and non-canonical splice sites variants such as last exonic nucleotide variants. These variants, often, do not cause an amino acid change but alter the snRNP proteins binding. We studied a big Italian family with Alport syndrome showing a clear dominant pattern of transmission with younger family members having only haematuria and older individuals presenting with End-Stage Kidney Failure (ESKF). Kidney biopsy showed the typical disease hallmarks. We deeply mined the data for SNV and CNV through exome sequencing on DNA from both peripheral blood samples and patients’ podocytes-lineage cells. We identified an already reported synonymous variant, c.765G>A (p.(Thr255Thr)), in the last exonic nucleotide of exon 13 of the *COL4A3* gene. Employing the patient’s podocytes we demonstrated that this variant results in exon skipping leading to an *in-frame* deletion of 28 amino acids without leaky effect. According to the pattern of transmission, to the kidney biopsy and to the exome data analysis we provided further evidence that autosomal dominant Alport syndrome is a well-defined clinical entity. We also confirmed the pathogenicity of the synonymous *COL4A3* variant for the first time demonstrating its role in a dominant pattern of transmission.

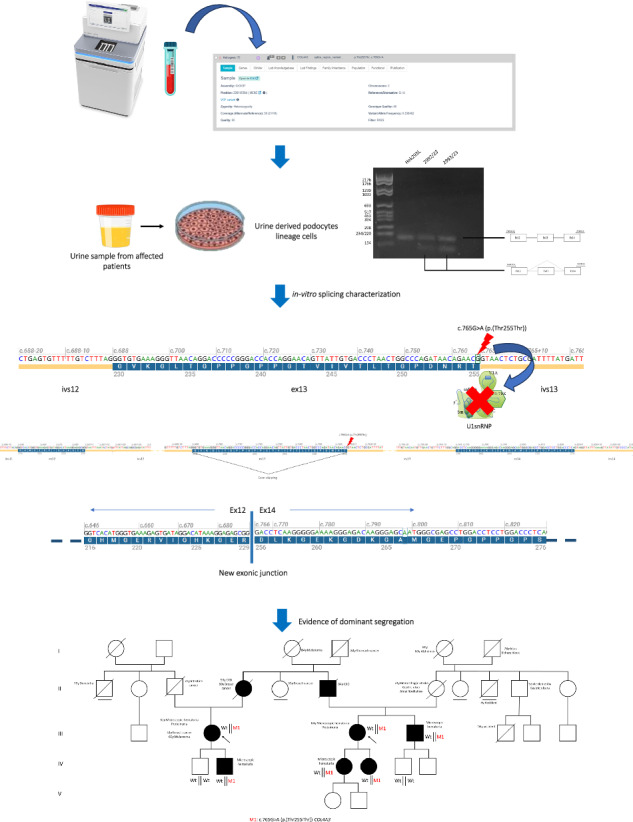

## Introduction

Alport syndrome (AS) is a rare genetic kidney disease characterised by mutations in COL4 genes, exhibiting a peculiar renal presentation with microscopic haematuria, proteinuria and a progression towards end-stage renal disease. Alongside these classic manifestations, the clinical presentation often includes sensorineural hearing loss (unilateral or bilateral) and visual manifestations such as lenticonus or macular flecks.

Over the years, four different patterns of inheritance have been described: X-linked (XLAS) [1–3], autosomal dominant (ADAS), autosomal recessive (ARAS) [4–8] and digenic (DAS) [9].

Often, in autosomal dominant disorder with clinical variability and reduced penetrance, it becomes challenging to classify some gene variants as pathogenic or likely pathogenic variants due to their presence in the heterozygous state. This hesitation stems from the observation that individuals harbouring a heterozygous pathogenic variant typically fail to manifest the complete spectrum of symptoms, likely attributable to the potential presence of additional variants in other *COL4* genes or in genes modulating the phenotype [10]. Additionally, a point of contention arises from the symptomatic overlap between ADAS and Thin Basement Membrane Nephropathy (TBMN), characterised by microscopic haematuria, generally normal kidney function and absence of proteinuria. However, in 2000, van der Loop and colleagues provided compelling evidence supporting the existence of ADAS. They identified a heterozygous mutation in the *COL4A3* gene within a large Alport Syndrome family from Northern Ireland [11], establishing the premise that AS, along with X-linked and ARAS forms, may encompass dominant forms that account for a significant proportion of cases (approximately around 30%) [4–13]. This number is likely to increase as we increasingly utilise NGS technologies to diagnose patients with milder cases of proteinuria or haematuria, which are indicative of ADAS forms. Moreover, NGS allows for more precise and comprehensive genetic analysis, facilitating the early detection and management of ADAS, even in individuals with subtle or less severe symptoms, enhancing our understanding of the disease’s prevalence and improving patient outcomes.

More recently, Prof. Roser Torra’s group published a moderately large study, albeit conducted in a limited patient sample cohort and within a single country, studying in deep the clinical manifestations of AD AS, raising nosological issues due the wide spectrum of clinical manifestations in these cases [14].

Over the past two decades, AS research has been primarily focused on the effects of exonic variants in collagen type IV. In the majority of papers substantial attention has been given to missense variants affecting the amino acid glycine, particularly within the collagenic domain. The goal has been to decipher the polarity changes and impacts of these variations on protein structure - a crucial step in understanding and in determining the role of these proteins within the collagenic heterotrimer.

The characterisation of splicing variants has lagged behind the exonic variants, mainly due to technical limitations and the clinical classification of these variants often remains uncertain. Aberrant COL4 splicing events are not only due to consensus splice site variations but also to non-canonical splice site variations, including those occurring in deep intronic regions or even substitutions in the last nucleotides of the exons [15]. Splicing variants could indeed have different impacts on kidney disease progression on the basis of aberrant splicing patterns observed. This underscores the need to elucidate the effects of splicing variants through cDNA analysis in order to unmask the impact on the transcript which can help define clinical progression, prognosis and familial recurrence risk.

However, as known, COL4 transcripts are not expressed on peripheral blood leucocytes and thus in-vitro minigene assays have so far helped to confirm most aberrant splicing patterns. We have recently demonstrated that transcript analysis after patients’ podocytes lineage cells establishment is the best tool to study the real effect of splicing variants on disease key-cells [16].

In this study, we present a wide Italian family with AS and a clear dominant pattern of transmission. Younger family members have only haematuria while older individuals developed ESKF around 65 years of age. Kidney biopsy displayed the typical disease hallmarks. Exome sequencing deep data analysis on DNA from both peripheral blood samples and patients’ podocytes-lineage cells only identified an already reported synonymous variant, c.765G>A (p.(Thr255Thr)), in the last exonic nucleotide of *COL4A3* exon 13. As previously reported by Deng H and colleagues, this variant leads to the complete skipping of the exon 13, resulting in an *in-frame* deletion involving the removal of 26 amino acids [17]. Taking advantage of the patient’s podocytes-lineage cells we confirmed that in the disease key-cells this variant results in exon skipping leading to an in-frame deletion of 28 amino acids. Deng’s study reported this variant as having a recessive mode of inheritance, as it co-segregates in a compound heterozygous state with a second variant in the *COL4A3* gene, although maternal side carrier manifested with haematuria. However, according to the pattern of transmission, to the kidney biopsy and to the exome data analysis we provided further evidence of the pathogenicity of this synonymous *COL4A3* variant in AS with an autosomal dominant pattern of transmission.

## Materials and methods

### Sample collection and diagnosis

Diagnosis of AS was established at the Medical Genetics Unit in Siena (Azienda Ospedaliera Universitaria Senese, AOUS). The family underwent genetic counselling, and blood samples were collected in EDTA-containing tubes for clinical exome analysis focusing on *COL4A3*, *COL4A4* and *COL4A5* mutational analysis.

Molecular testing was extended to relatives of patients with a germline mutation. Urine samples were collected in Urine Container Plus 100 mL for urine cell isolation. Informed consent was obtained from all adult patients for diagnostic and research purposes and for urine samples collection.

The study was approved by the Azienda Ospedaliera Universitaria Senese Ethics Committee.

### Clinical exome sequencing sample preparation and analysis

Sample preparation was performed following the Illumina Nextera Flex for Enrichment manufacturer protocol. The workflow uses a bead-based transposome complex to tagment genomic DNA, which is a process that fragments DNA and then tags the DNA with adaptor sequences in one step, preparing them for sequencing. The exome sequencing analysis was performed on the Illumina NovaSeq 6000 System (Illumina San Diego, CA, USA) according to the NovaSeq 6000 System Guide. Reads were mapped against the hg19 reference genome by using the Burrow-Wheeler aligner BWA [18]. After saturation with input DNA, the bead-based transposome complex fragments a set number of DNA molecules. This fragmentation provides flexibility to use a wide DNA input range to generate normalised libraries of consistent tight fragment size distribution. After sequencing the data were analysed for SNV and CNV identification with the support of dedicated alignment algorithms for SNVs and the employment of EXCAVATOR2 for CNVs. Variants annotation and prioritisation were performed using eVai software (enGenome) side by side of a consulting of the online variant databases (ClinVar and LOVD). An extensive description of the procedure and data analysis is available in Supplementary Materials.

### Urine-derived podocytes-lineage cells isolation

To establish a patient-derived cell system resembling podocytes’ physiological conditions, we adapted a protocol for isolating cells from urine samples of AS patients [16]. Urine was processed within 4 h of collection to prevent hyper acidification. Samples were centrifuged at 2400 rpm for 10 min, washed with 10 mL of Washing Buffer, and centrifuged again. The pellet was then resuspended in primary medium and plated onto gelatin-treated culture plates. The primary medium consisted of DMEM/high glucose and Ham’s F12 mix supplemented with foetal bovine serum (FBS), antibiotics, antifungal agent, and renal epithelial cell growth medium Single Quote kit supplements. After 3 days, a portion of the medium was replaced with RE/MC expansion medium to select for podocyte-lineage cell growth. The expansion medium was a combination of RE cells basal medium and MC medium, supplemented with growth factors. Proliferation medium was changed daily until two distinct groups of colonies were observed: one with regular appearance and cobblestone-like morphology, and another with higher proliferation rate. Cells were split around 9–12 days after plating.

Once isolated the cells with high proliferation rate were characterised by means of RT-PCR analysis that revealed *COL4A3*, *COL4A4* and *COL4A5* expression associated with the expression of Podoplanin (*PDPN*), marker involved in shaping podocytes membrane. In addition, transcription factors such as *PAX2* and *PAX8* and a marker belonging to tight junction family, Occludin (*OCLN*), were expressed in the first replicative phase, in the terminal cultures. Immunofluorescence detected the expression of podocytes differentiation markers such as Podocalyxin (*PODXL*). These results confirm that our cells belong to the podocytes lineage although they are not completely differentiated.

### Urine-derived podocyte-lineage cells DNA isolation

Total DNA was extracted, starting from cell pellet by two wells of a six-well culture dish, using the QIAamp DNA Micro Kit (QIAGEN R, Hilden Germany) in accordance with the manufacturer’s protocol. DNA was eluted with 40 μl of RNase FreeWater.

The concentration of DNA was determined by using the Qubit™ 4 Fluorometer (Thermo Fisher Scientific R, Waltham, Massachusetts, United States) in accordance with the manufacturer’s protocol.

### Urine-derived podocyte-lineage cells RNA isolation and cDNA synthesis

Total RNA was extracted, starting from cell pellet, by two wells of a six-well culture dish, using the RNeasy Mini Kit (QIAGEN R, Hilden Germany) in accordance with the manufacturer’s protocol. RNA was eluted with 40 μl of RNase FreeWater.

The concentration of RNA was determined by using a Nanodrop 2000 Spectrophotometer (Thermo Fisher Scientific R, Waltham, Massachusetts, United States). A260/A280 ratios were also calculated by the instrument for each sample.

cDNA was synthesised from total RNA using QuantiTect Reverse Transcription Kit (QIAGEN R), in accordance with the manufacturer’s instructions, starting from 1μg of total RNA per reaction.

## Case report

### Patient 1

Patient 1 (5813/21) refers to a 62 years old female patient who presented with proteinuria and microscopic haematuria. Laboratory findings included an estimated glomerular filtration rate (eGFR) of 68.5 mL/min. Patient 1 patient decided not to undergo a kidney biopsy. A pure tone audiogram was conducted, revealing no abnormalities.

The patient was diagnosed with breast cancer at 51 years of age; she underwent surgery, chemotherapy and radiation therapy. Subsequently, at the age of 60, she was diagnosed with melanoma and underwent surgery.

**Fig. 1:**
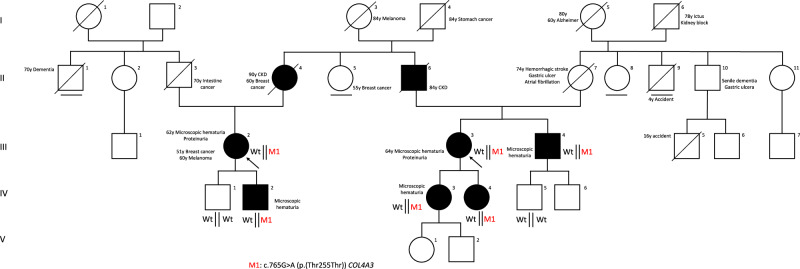
Family pedigree with evident dominant segregation. The pedigree of the family showed a typical autosomal dominant segregation of the investigated variant.

The patient’s family history revealed the presence of chronic kidney disease (CKD) in her mother around 70 years of age and haematuria in her son. In Fig. 1, Patient 1 is represented as III:2

### Patient 2

Patient 2 (472/23) is the maternal cousin of the patient presented as patient 1 (III:3). She is a 64 years old female patient. Like Patient 1, she also exhibited symptoms of proteinuria and microscopic haematuria with reduced renal function. The patient reported that proteinuria was initially observed when she was 15 years old. Laboratory findings included an eGFR of 57.4 mL/min, 24-h Urine Protein of 0.40 g/24 h, and urine creatinine of 45.8 mg/dL (see Supplementary Material). Furthermore, these laboratory parameters justified the need for conducting a renal biopsy.

**Fig. 2:**
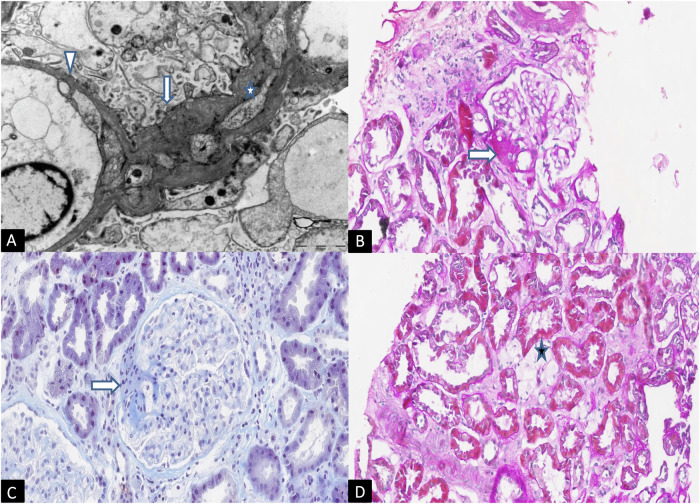
Electron microscopy, Periodic acid–Schiff stain (PAS) and Acid Fuchsin Orange G (AFOG) staining on kidney biopsy sections. **A** Electron microscopy, UAR, x8000. Extremely thin basal glomerular membrane (GMB) (arrow-head) with regular contour and effacement of foot processes (such lesion was widespread in the glomerular loops). Thickening and irregular appearance of GBM with fragmenting (asterisk), newly formed membrane, reduplication and focal scalloping of foot processes (arrow). These lesions were focal in the glomerular loops. **B** Pas, x400.Focal segmental glomerulosclerosis (FSG) with capsular adhesion (arrow). **C** AFOG, x400. The same lesion (FSG) in the next section (arrow). **D** Pas, x400 Accumulation of lipids is frequent in interstitial cells such as macrophages (asterisk).

Patient 2 underwent kidney biopsy for histopathological diagnosis in accordance with the Kidney Disease Improving Global Outcomes (KDIGO) good clinical practice guidelines [19]. Optical and electron microscopy examination of the core biopsy revealed histologic features and ultrastructural alterations suggestive of AS (Fig. 2).

No comorbidities were reported.

The patient’s family history revealed the presence of CKD in her father, as well as microscopic haematuria in both of her daughters and in her brother.

### Other family members clinical features

Patient IV:1, aged 37, reported no microscopic haematuria. Laboratory findings included a serum creatinine level of 1.04 mg/dL and an eGFR of 91.3 ml/min. Urine sediment analysis showed 6 RBC/μL (normal value < 20 RBC/μL). No proteinuria was detected.

His brother, patient IV:2 (6535/23), aged 31, reported microscopic haematuria since infancy. While no proteinuria was detected, urine sediment analysis showed 132 RBC/μL.

Patient IV:3 (6537/23), aged 36, reported microscopic haematuria since infancy. Unfortunately, the patient didn’t perform any laboratory analysis. Thus, no data about creatinine nor urine sediment is available.

Her sister, patient IV:4 (6539/23), aged 32, didn’t report microscopic haematuria. Laboratory findings included a serum creatinine level of 0.64 mg/dL and an eGFR of 117.2 ml/min and. Urine sediment analysis showed 21 RBC/μL. No proteinuria was detected.

Patient III:4 (6927/23), aged 59, reported microscopic haematuria since infancy and bilateral hearing impairment few years back. Laboratory findings included a serum creatinine level of 0.88 mg/dL, an eGFR of 94.0 ml/min and urine protein levels of 50 mg/dl. Urine sediment analysis showed 84 RBC/μL.

His son, patient IV:5 (6929/23), aged 27, didn’t report microscopic haematuria. Laboratory findings included a serum creatinine level of 0.81 mg/dL and an eGFR of 121.8 ml/min. Urine sediment analysis showed 17 RBC/μL. No proteinuria was detected.

No information about his brother IV:6 was available.

No comorbidities were reported in any of these patients.

The clinical features, medications and comorbidities of patient 1, patient 2 and involved family members are summarised in Table 1.

**Table 1:** Patient's clinical and molecular characteristics.

Family ID	Position in the pedigree	Sex	Age (years)	Mutated Gene	Mutated nucleotide	Mutated aminoacid	CADD	Microscopic hematuria	Macroscopic hematuria	Proteinuria	Creatinine	eGFR	Hearing impairment	Visual impairment	Nephrotic Syndrome	Kidney Failure	Kidney Transplantation	Medications
5813/21	III:2	F	64	*COL4A3*	c.765G>A	(p.(Thr255Thr))	25.2	Yes	No	Yes	0,90 mg/dL	68,5 mL/min*	No	No	No	No	No	No
472/23	III:3	F	62					Yes	No	Yes	1,03 mg/dL	57,4 mL/min	No	No	No	No	No	Ramipril 10mg, Dapaglifozin 10mg
6927/23	III:4	M	59					Yes	No	Yes (0,5mg/dL)	0,88 mg/dL	94 mL/min	Yes	No	No	No	No	Ramipril 2,5mg
6535/23	IV:2	M	31					Yes	No	No	Na	Na	No	No	No	No	No	No
6537/23	IV:3	F	36					Na	No	No	Na	Na	No	No	No	No	No	No
6539/23	IV:4	F	32					Yes	No	No	0,64 mg/dL	118,1 mL/min	No	No	No	No	No	No
6533/23	IV:1	M	37	/	/	/	/	No	No	No	1,04 mg/dL	91,3 mL/min	No	No	No	No	No	No
6929/23	IV:5	M	27					No	No	No	0,81 mg/dL	121,8 mL/min	No	No	No	No	No	No

*Stages of chronic kidney diseases eGFR reference value in accordance with Levey AS et al.

90+ Normal eGFR, normal renal function or minimally impaired.

60–89 eGFR slightly lower than normal.

30–59 eGFR lower than normal.

15–29 eGFR much lower than normal.

<15 Kidney failure.

## Results

### c.765G>A (p.(Thr255Thr)) COL4A3 variant identification

**Fig. 3:**
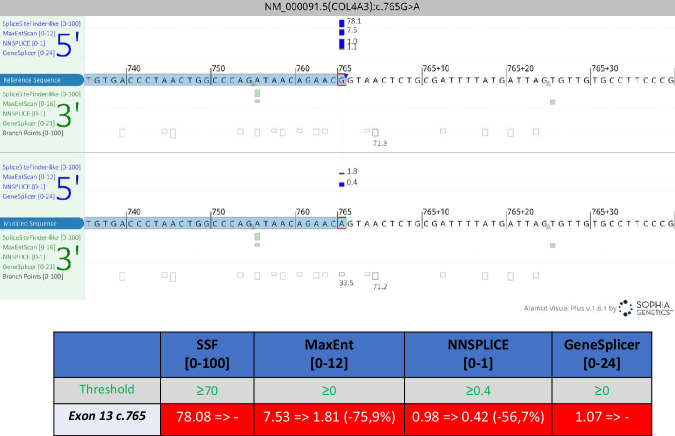
In-silico splicing variant prediction effect. Bio-Prediction of splice variations using Alamut™ Visual Plus—Variant Annotation and Analysis Software.

Both patients underwent blood withdrawal and urine samples collection. Clinical exome sequencing performed on DNA from peripheral blood samples of patient 1 and 2 allowed us to identify the c.765G>A transition located in the last exonic nucleotide of exon 13. This variation does not result in an amino acid substitution but in a synonymous variant p.Thr255Thr. However, this variant has a significant impact on the splicing process due to its peculiar localisation, resulting in a complete abrogation of the canonical donor splice site for SpliceSiteFinderLike and GeneSplicer and a substantial reduction of affinity for NNSPLICE and MaxEntScan. In particular, the variation for the MaxEntScan and NNSPLICE prediction programmes is −75.9% and −56.7% respectively, defining it as a high impact variation unquestionably (Fig. 3). This variant is also reported on SpliceValut database software (https://kidsneuro.shinyapps.io/splicevault/) [20] and was added to the *COL4A3*-related LOVD database (https://databases.lovd.nl/shared/genes/COL4A3).

### Clinical exome sequencing on DNA isolated from urine-derived podocytes lineage cells

In order to evaluate the presence of a second somatic hit in the renal tissue, not revealed from genetic analysis performed on DNA from peripheral blood sample, a clinical exome sequencing on DNA from urine-derived podocytes-lineage cells was performed on Patient 1 and Patient 2.

With the exception of the germinal p.Thr255Thr variant, no other variants in *COL4A3* and in the other COL4 genes (*COL4A4* and *COL4A5*) - both pathogenic or uncertain—were detected in both samples allowing us to exclude a recessive or digenic pattern of transmission.

### Search for modifier genes

In order to exclude the contribution of other genes as modifiers we deeply mined exonic data from both peripheral blood samples and urine-derived podocyte-lineage cells to search for rare heterozygous and homozygous variants with clear pathogenic or likely pathogenic effects. The analysis was mainly focused on a set of genes already reported in literature [21–23] as modifier or potential modifier in AS (*ALB, CFHR5, DDR1, FMN1, HBEGF, ITGA1, ITGA2, ITGB6, KIRREL2, LAMA5, LAMB2, MMP9, MYH9, MYO1E, NPHS1, NPHS2, SOSTDC1, SPP1, SYNPO, TP53*). However, no variants were found in any of these genes among the screened family members.

Given that no other pathogenic or likely pathogenic variants were identified during this additional analysis, it is likely that the only variant responsible for the observed phenotypic profile in this family is the p.(Thr255Thr) *COL4A3* variant. In according to the molecular data, the family segregation and the slowly progressive kidney damage we met the ADAS criteria.

### Segregation analysis in family members

In order to unquestionably demonstrate a dominant pattern of transmission, we performed segregation analysis by the means of Sanger sequencing in the two sons of Patient 1, in the two daughters of Patient 2, in the brother of Patient 2 and ultimately in one of his sons. The variant was present in heterozygous state in the symptomatic family members, segregating with the disease.

### c.765G>A (p.(Thr255Thr)) COL4A3 transcript analysis

**Fig. 4:**
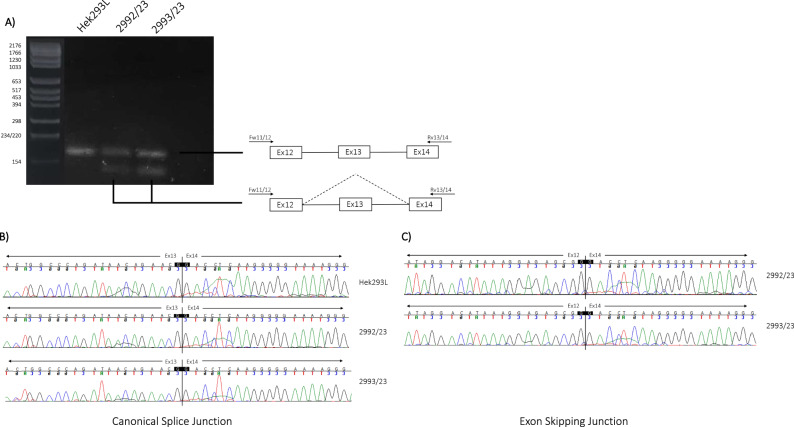
c.765G>A (p.(Thr255Thr)) variant pathogenic role definition by transcript analysis. **A** RT-PCR was performed on the endogenous *COL4A3* gene using RNA from patients-derived podocytes-lineage cells. Diagrams beside each PCR product represent the structure of detected transcripts, with arrowheads indicating the location of primers (left side). **C** The transcript analysis allows us to conclude about the pathogenic effect of the variant leading to the exon 13 skipping, resulting in an in-frame deletion of the entire exon and the generation of a new exonic junction between exon12 and exon 14 (**B**).

In the reported family, affected members harboured a variant in the last nucleotide of the exon 13 of the *COL4A3* gene c.765G>A (p.(Thr255Thr)). Despite being a synonymous variant, its localisation has a significant effect on the splicing process, in particular by altering the consensus sequence of the canonical 5′splice site (5′SS). As previously demonstrated by Deng et al. [17], such a variant hampering U1snRNA binding at the 5′SS and thus disrupting the exon definition process, leads to an exon skipping event. To confirm that this event occurs also in our family, transcript analysis was performed on mRNA isolated from urine-derived podocytes-lineage cells of both the proband and her cousin (Patient 1 and Patient 2). A glomerulus-derived control cell line, Hek293L, was used as control. Reverse Transcription Polymerase Chain Reaction (RT-PCR) performed using a pair of primers spanning exons 11–12 and exons 14–15 junctions revealed the presence of a lower band in all tested family members, which was absent in the control sample. Sequencing analysis confirmed that the lower PCR product lacked exon 13, indicating an exon skipping event (Fig. 4).

Our results confirmed that in patients’ key-cells, the Thr255Thr variant is able to trigger aberrant splicing events resulting in an exon skipping of the entire exon 13 leading to a shorter protein (1644aa compared to the 1670aa longer wild-type protein) as a consequence of 78 nucleotides removal in the alternative transcript sequence.

## Conclusion and discussion

Among causative variants in AS, *COL4A3*/*COL4A4*/*COL4A5* splicing variants account for a significant proportion of the total variants detected, being not less than 25–30% [13, 15, 24]. The number of reported splicing variants continues to increase due to the advances in molecular analysis and the availability of more precise in-vitro platforms to perform genetic characterisation of their effect on the transcript. Therefore, in cases where the segregation of a splicing variant is evident, it is highly recommended to conduct transcript analysis on mRNA. Furthermore, even exonic nucleotide substitutions can cause aberrant pre-mRNA splicing effects, especially when they are located near canonical splice sites. In such cases, transcript analysis is crucial, especially for patients who harbour variants in specific regions (start or end of each exons) or display an unexpected phenotype.

The variant described in our family refers to a non-amino acid substitution (synonymous variant). Deng and colleagues previously reported the same variant in trans with a second missense variant of the *COL4A3* gene, concluding for its role in a recessive pattern of transmission. In the same work they have shown that this variant leads to an exon skipping with the consequent removal of 26 amino acids without altering the reading frame [17]. Here, we have confirmed that in patients’ disease key-cells this variant determines an aberrant splicing event with no leaky effect, and we have contributed to incontrovertible re-classify this variant as pathogenic in accordance with the ACMG/AMP criteria.

While Deng and colleagues proposed an autosomal recessive segregation (ARAS), previous reports from the Centre for Mendelian Genomics, University Medical Centre Ljubljana, Bioscientia Institut für Medizinische Diagnostik GmbH, Sonic Healthcare and Fulgent Genetics suggested an autosomal dominant inheritance for this variant. However, all these reports lacked a clear functional characterisation of the variant c.765G>A. Taking advantage of a thorough exome sequencing analysis in a wide Italian family in which younger individuals display only haematuria and older ones kidney failures and in which affected individuals have suggestive kidney biopsy, for the first time we have provided compelling evidence of the role of the identified synonymous variant in autosomal dominant AS. It’s important to note that the older relatives who suffered from kidney failure have passed away and weren’t included in the genetic analysis; nonetheless they were obligated carriers (Fig. 1). The unique support provided by the urine-derived podocyte-lineage cells allowed us not only to extract RNA for re-characterising the effect of the variant but also to exclude the possibility of a second somatic hit in the affected tissue.

Our work strengthens the recent consensus about the existence of broader phenotype of ADAS and the need not to consider it as benign condition. Moreover, it is important to point out that carriers need to be considered for clinical screening based on the evidence. It is in line with recent data according to which about 20-30% of patients with ADAS due to *COL4A3* and *COL4A4* pathogenic variants with persistent microscopic haematuria develop CKF, 15% develop ESKF with a median age of onset of 50 years, and about 10% have extrarenal features [25]. Indeed, our family history supports the notion that ESKF can manifest late in life in ADAS, potentially leading to underestimation of kidney disease progression. Therefore, a tailored follow-up is necessary for individuals with heterozygous *COL4* variants showing clear autosomal dominant transmission especially when there is a positive family history of CKF and/or consideration for living donor transplantation, at this may increase the risk of developing CKF. The follow-up should include regular nephrological assessments, and laboratory tests to promptly detect renal function decline and adjust pharmacological regimens. Initial therapy with ACE-inhibitors has been recommended for family members with identified familial variants. A therapy with ACE-inhibitor has indeed been associated with a later onset of ESKF and also a longer life expectancy [26].

No definitive cures are currently available for AS and pharmacological approaches aim to delay the progression of clinical symptoms. In line with these observations, a CRISPR/Cas9 system [27] able to target and correct this nucleotide variation could correct the genetic defect and potentially restore the canonical splice site of exon 13, preventing exon skipping.

## Supplementary information

Supplementary Materials

Supplementary Table 1

Supplementary Figure

Supplementary Figure legends

## Data Availability

The datasets generated and/or analysed during the current study are available from the corresponding author upon reasonable request.
