# Correction: Slowly progressive autosomal dominant Alport Syndrome due to *COL4A3* splicing variant

**DOI:** 10.1038/s41431-024-01736-2

**Published:** 2024-11-25

**Authors:** Sergio Daga, Lorenzo Loberti, Giulia Rollo, Loredaria Adamo, Olga Lorenza Colavecchio, Giulia Brunelli, Kristina Zguro, Sergio Antonio Tripodi, Andrea Guarnieri, Guido Garosi, Romina D’Aurizio, Francesca Ariani, Rossella Tita, Alessandra Renieri, Anna Maria Pinto

**Affiliations:** 1 Medical Genetics, University of Siena, Siena, Italy; 2 Med Biotech Hub and Competence Center, Department of Medical Biotechnologies, University of Siena, Siena, Italy; 3 Genetica Medica, Azienda Ospedaliero-Universitaria Senese, Siena, Italy; 4 Institute of Informatics and Telematics, CNR, Pisa, Italy; 5 Department of Pathology, AOUS, Siena, Italy; 6 Department of Medical Sciences, Nephrology, Dialysis and Transplantation Unit, University Hospital of Siena, Siena, Italy

**Keywords:** Alport syndrome, Genetics research

Correction to: *European Journal of Human Genetics* 10.1038/s41431-024-01706-8, published online 19 October 2024

Table 1 was missing from this article; the figure should have appeared as shown below.

**Table 1:** Patient’s clinical and molecular characteristics.

Family ID	Position in the pedigree	Sex	Age (years)	Mutated Gene	Mutated nucleotide	Mutated aminoacid	CADD	Microscopic hematuria	Macroscopic hematuria	Proteinuria	Creatinine	eGFR	Hearing impairment	Visual impairment	Nephrotic Syndrome	Kidney Failure	Kidney Transplantation	Medications
5813/21	III:2	F	64	*COL4A3*	c.765G>A	(p.(Thr255Thr))	25.2	Yes	No	Yes	0,90 mg/dL	68,5 mL/min*	No	No	No	No	No	No
472/23	III:3	F	62					Yes	No	Yes	1,03 mg/dL	57,4 mL/min	No	No	No	No	No	Ramipril 10mg, Dapaglifozin 10mg
6927/23	III:4	M	59					Yes	No	Yes (0,5mg/dL)	0,88 mg/dL	94 mL/min	Yes	No	No	No	No	Ramipril 2,5mg
6535/23	IV:2	M	31					Yes	No	No	Na	Na	No	No	No	No	No	No
6537/23	IV:3	F	36					Na	No	No	Na	Na	No	No	No	No	No	No
6539/23	IV:4	F	32					Yes	No	No	0,64 mg/dL	118,1 mL/min	No	No	No	No	No	No
6533/23	IV:1	M	37	/	/	/	/	No	No	No	1,04 mg/dL	91,3 mL/min	No	No	No	No	No	No
6929/23	IV:5	M	27					No	No	No	0,81 mg/dL	121,8 mL/min	No	No	No	No	No	No

*Stages of chronic kidney diseases eGFR reference value in accordance with Levey AS et al.

1 90+ Normal eGFR, normal renal function or minimally impaired.

2 60–89 eGFR slightly lower than normal.

3 30–59 eGFR lower than normal.

4 15–29 eGFR much lower than normal.

5 <15 Kidney failure.

The original article has been corrected.

