# Supplementary material for: Slowly progressive autosomal dominant Alport Syndrome due to COL4A3 splicing variant
Source: Eur J Hum Genet. 2024 Oct 19;33(4):461–7. doi: 10.1038/s41431-024-01706-8 (PMC11985956; doi:10.1038/s41431-024-01706-8)
Supplement: Supplementary file 1 — Supplementary Materials [file 41431_2024_1706_MOESM1_ESM.docx]

*Clinical Exome Sequencing sample preparation and analysis*

Sample preparation was performed following the Nextera Flex for Enrichment manufacturer protocol. The workflow uses a bead-based transposome complex to tagment genomic DNA, which is a process that fragments DNA and then tags the DNA with adapter sequences in one step. After saturation with input DNA, the bead-based transposome complex fragments a set number of DNA molecules. This fragmentation provides flexibility to use a wide DNA input range to generate normalized libraries of consistent tight fragment size distribution. Following tagmentation, a limited-cycle PCR adds adapter sequences to the ends of a DNA fragment. A subsequent target enrichment workflow is then applied. Following pooling, the double stranded DNA libraries are denatured and biotinylated TruSight One Expanded Oligonucleotide probes are hybridized to the denatured library fragments. After hybridization, Streptavidin Magnetic Beads (SMB) then capture the targeted library fragments within the regions of interest. The captured and indexed libraries are eluted from beads and further amplified before sequencing. The exome sequencing analysis was performed on the Illumina NovaSeq 6000 System (Illumina San Diego, CA, USA) according to the NovaSeq 6000 System Guide. Reads were mapped against the hg19 reference genome by using the Burrow-Wheeler aligner BWA [S1]. Variant calling was obtained using an in-house pipeline which takes advantage of the GATK Best Practices workflow [S2].

Alongside SNVs and INDELs, the clinical exome sequencing analysis is completed with the processing of the CNVs reported in VCF files and classify them according to these international guidelines. In this context, technical standards for CNV classification have been recently published by ACMG and ClinGen [S3], introducing semiquantitative point-based scoring metrics as support for pathogenicity assessment. This guarantees a fundamental advancement in terms of complexity reduction of the CNV analysis, since manual interpretation is time consuming and error-prone. The pipeline for CNV analysis is based on EXCAVATOR2, a read count-based tool that exploits all reads produced by the exome experiments to detect CNVs with genome-wide resolution by integrating the analysis of In-target and Off-target reads [S4]. Specifically, for both test subjects, the clinical exome was prepared using the Illumina TruSight One Expanded panel on the hg19 reference genome. SNVs were called using the Illumina DRAGEN platform (v4.2), while CNV detection was performed with an optimised Singularity-based version of EXCAVATOR2 (<https://github.com/ctglab/exca2sing>), using a W=50K length for off-target regions. In the CNVs calling procedure, for each sample under investigation, we set up all samples from the same batch and of the same concordance sex as the pool of controls to account for batch effects and to be able to detect CNVs on the X chromosome More precisely for sample 472/23, 58 women from the same batch were used as controls, and for sample 5813/21, 25 women from the same batch. The detected CNVs were divided into three categories based on the call probability, as a proxy for call quality, provided by EXCAVATOR2: "PASS” if the probability of a call was higher than 0.8, “Medium Quality” if the probability of call is between 0.5 and 0.8, and “Low Quality” if the probability of call is less than 0.5. The .vcf files produced by EXCAVATOR2 were merged with those from the DRAGEN output and uploaded on eVAI for the annotation and prioritisation phase.

CES data were filtered using eVai (enGenome) software. Variant’s prioritization was obtained by using increasingly enlarged filters: i) genes (*COL4A3*, *COL4A4* and *COL4A5*) and ii) phenotype (using HPO terms: microscopic, proteinuria). In order to find PVs, we focused our attention on rare variants (minor allele frequency, MAF <0,01). Frameshift, stop gain and splice site variants were prioritized as pathogenic. Missense variants were predicted to be damaging by CADDphred prediction tools (score ≥25) and splice site variants by MaxEnt Scan Tool. The following public databases were used for interpretation of the variants: ClinVar (http[s:www.ncbi.n](http://www.ncbi.nlm.nih.gov/clinvar/))lm[.nih.gov/clinvar/)](http://www.ncbi.nlm.nih.gov/clinvar/)) and LOVD (hptts://database.lovd.nl/shared/genes).

References:

S1. Li H, Durbin R. Fast and accurate long-read alignment with Burrows-Wheeler transform. Bioinformatics. 2010; 26(5):589-95

S2. Poplin R, Ruano-Rubio V, DePristo MA, Fennell TJ, Carneiro MO, Van der Auwera GA, et al. Scalingaccurate genetic variant discovery to tens of thousands of samples. bioRxiv. 2018.

S3. Riggs ER, Andersen EF, Cherry AM, Kantarci S, Kearney H, Patael A, et al. Technical standards for the interpretation and reporting of constitutional copy-number variants: a joint consensus recommendation of the American College of Medical Genetics and Genomics (ACMG) and the Clinical Genome Resource (ClinGen). Genet Med. 2020; 22(2):245-257.

S4. D'Aurizio R, Pippucci T, Tattini L, Giusti B, Pellegrini M, Magi A. Enhanced copy number variants detection from whole-exome sequencing data using EXCAVATOR2. 2016; 16;44(20):e154.
