# Supplementary figures and images for: Slowly progressive autosomal dominant Alport Syndrome due to COL4A3 splicing variant
Source: Eur J Hum Genet. 2024 Oct 19;33(4):461–7. doi: 10.1038/s41431-024-01706-8 (PMC11985956; doi:10.1038/s41431-024-01706-8)

Patient 2

A

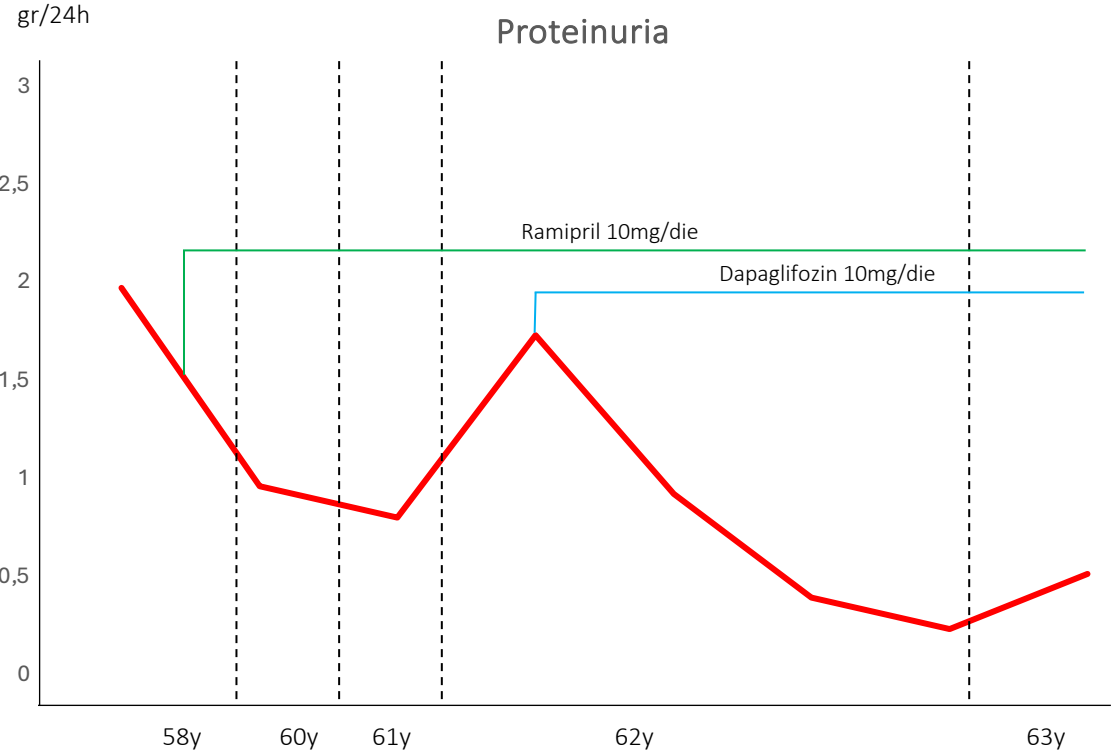

B

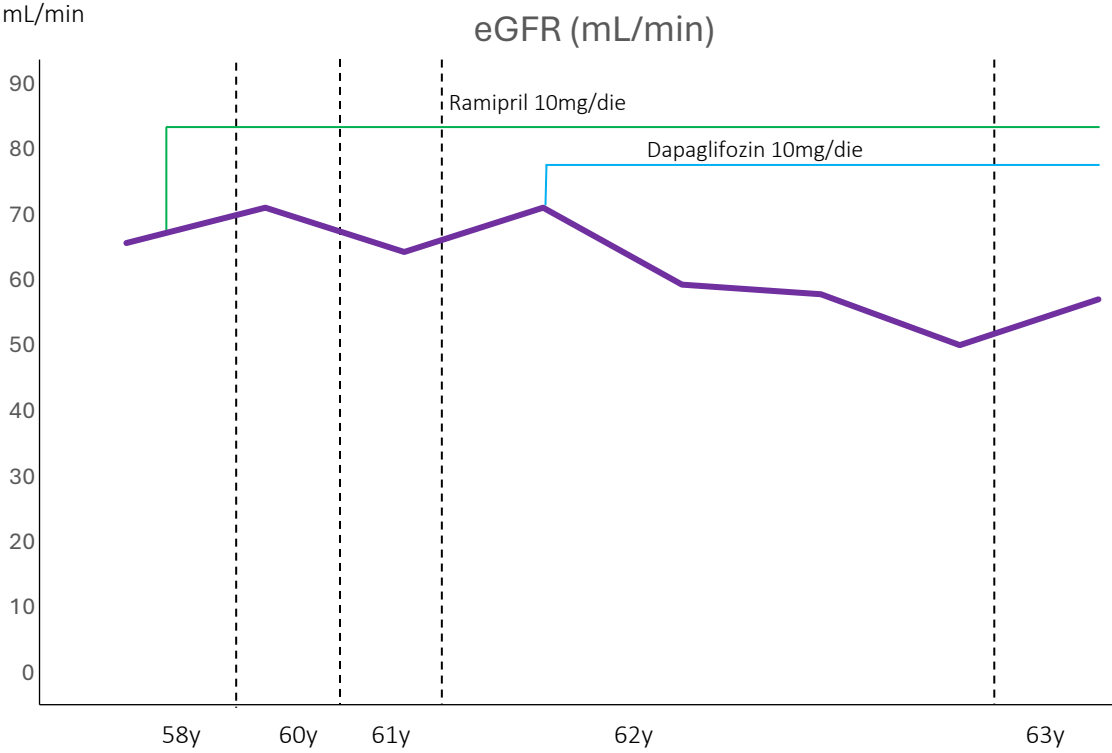

Supplement: Supplementary file 3 — Supplementary Figure [file 41431_2024_1706_MOESM3_ESM.pdf]
