# Supplementary material for: Slowly progressive autosomal dominant Alport Syndrome due to COL4A3 splicing variant
Source: Eur J Hum Genet. 2024 Oct 19;33(4):461–7. doi: 10.1038/s41431-024-01706-8 (PMC11985956; doi:10.1038/s41431-024-01706-8)
Supplement: Supplementary file 4 — Supplementary Figure legends [file 41431_2024_1706_MOESM4_ESM.docx]

**Supplementary Figure 1.**  Trend of proteinuria and eGFR of Patient 2 in a 5-years timespan. The start of ACEi and SGLT2i regimens is indicated with green and blue lines, respectively.
