# Supplementary material for: Slowly progressive autosomal dominant Alport Syndrome due to COL4A3 splicing variant
Source: Eur J Hum Genet. 2024 Oct 19;33(4):461–7. doi: 10.1038/s41431-024-01706-8 (PMC11985956; doi:10.1038/s41431-024-01706-8)
Supplement: Supplementary file 2 — Supplementary Table 1 [file 41431_2024_1706_MOESM2_ESM.pdf]

Supplementary Table 1. Patient's clinical characteristics comparison with Deng reported case

Family ID	Position in the pedigree	Sex	Age (years)	Mutated Gene	Mutated nucleotide	Mutated aminoacid	CADD	Age of Onset	Microscopic hematuria	Macroscopic hematuria	Proteinuria	Creatinine	eGFR	Hearing impairment	Visual impairment	Nephrotic Syndrome	Kidney Failure	Kidney Transplantation	Medications
5813/21	III:2	F	64	*COL4A3*	c.765G>A	(p.(Thr255Thr))	25,2	Na	Yes	No	Yes	0,90 mg/dL	68,5 mL/min	No	No	No	No	No	No
472/23	III:3	F	62					15	Yes	No	Yes	1,03 mg/dL	57,4 mL/min	No	No	No	No	No	Ramipril 10mg, Dapaglifozin 10mg
Deng et al.	III:1	M	25	*COL4A3*	c.765G>A	(p.(Thr255Thr))	25,2	3	Yes	Yes	Yes	1,85 mg/dL	49,6 mL/min	No	No	No	No	No	Na
					c.4664C>T	(p.(Ala1555Val))	23,6												
